# Pancreaticoduodenectomy with reconstructing blood flow of the gastric conduit after esophagectomy with concomitant celiac axis stenosis: a case report

**DOI:** 10.1186/s40792-020-01019-0

**Published:** 2020-10-08

**Authors:** Masaaki Minagawa, Hirofumi Ichida, Ryuji Yoshioka, Yu Gyoda, Tomoya Mizuno, Hiroshi Imamura, Yoshihiro Mise, Hidehiko Yoshimatsu, Yuki Fukumura, Kota Kato, Yoshiaki Kajiyama, Akio Saiura

**Affiliations:** 1 Department of Hepatobiliary-Pancreatic Surgery, Juntendo University, Graduate School of Medicine, 2-1-1, Hongo, Bunkyo-ku, Tokyo, 113-8421 Japan; 2 Department of Plastic and Reconstructive Surgery, Cancer Institute Hospital, Japanese Foundation for Cancer Research, 3-8-31 Ariake, Koto-ku, Tokyo, 135-8550 Japan; 3 Department of Human Pathology, Juntendo University, Graduate School of Medicine, 2-1-1, Hongo, Bunkyo-ku, Tokyo, 113-8421 Japan; 4 Department of Anatomy and Life Structure, Juntendo University, Graduate School of Medicine, 2-1-1, Hongo, Bunkyo-ku, Tokyo, 113-8421 Japan; 5 Department of Esophageal and Gastroenterological Surgery, Juntendo University, Graduate School of Medicine, 2-1-1, Hongo, Bunkyo-ku, Tokyo, 113-8421 Japan

**Keywords:** Pancreaticoduodenectomy, Gastric conduit-preserving, Esophagectomy, Celiac artery stenosis, Microvascular reconstruction, Dorsal pancreatic artery

## Abstract

**Background:**

Pancreaticoduodenectomy after esophageal resection is technically difficult, because blood flow of the gastric conduit should be preserved. Celiac axis stenosis (CAS) is also a problem for pancreaticoduodenectomy, because arterial blood supply for the liver comes mainly through the collateral route from the superior mesenteric artery (SMA) via the gastroduodenal artery (GDA). Herein, we report the case of a patient with pancreatic head cancer who underwent a pancreaticoduodenectomy after esophagectomy with concomitant CAS.

**Case presentation:**

A 76-year-old man with pancreatic head cancer was referred to our department. He had a history of esophagectomy with retrosternal gastric conduit reconstruction for esophageal cancer. Computed tomography showed severe CAS and a dilated collateral route between the SMA and the splenic artery (SPA). We prepared several surgical options depending on the intraoperative findings, and performed radical pancreaticoduodenectomy with concomitant resection of the distal gastric conduit. The right gastroepiploic artery (RGEA) of the remnant gastric conduit was fed from the left middle colic artery (MCA) with microvascular anastomosis. Despite CAS, when the GDA was dissected and clamped, good blood flow was confirmed, and the proper hepatic artery did not require reconstruction. The patient was discharged on postoperative day 90.

**Conclusions:**

We successfully performed radical pancreaticoduodenectomy after esophagectomy with concomitant CAS, having prepared multiple surgical options depending upon the intraoperative findings.

## Background

Pancreaticoduodenectomy is a radical treatment for pancreatic head cancer. It is technically difficult to perform pancreaticoduodenectomy in patients who have undergone subtotal esophagectomy in the past, especially if followed by reconstruction of the gastric conduit. The blood flow to the gastric conduit is usually supplied mainly by the gastroduodenal artery (GDA) via the right gastroepiploic artery (RGEA) which is divided during pancreaticoduodenectomy.

Celiac axis stenosis (CAS) is another hurdle for undergoing pancreaticoduodenectomy, and is caused by external compression or internal occlusion [1–7]. In many cases of CAS, the gastroduodenal artery (GDA) is the main collateral pathway from the superior mesenteric artery (SMA) to the celiac artery system through the common hepatic artery (CHA) [8, 9]. In CAS, the division of the GDA during pancreaticoduodenectomy can cause an ischemic threat, not only to the liver, stomach, and remnant pancreas, but also to the gastrojejunal, hepaticojejunal, and pancreaticojejunal anastomoses [10].

Precise preoperative diagnosis is difficult, because pancreatic cancer exhibits local invasive growth. Therefore, we are often obliged to make decisions of vascular resection and reconstruction intraoperatively. As such, based on the preoperative CT, multiple surgical choices should be prepared for different situations.

Here, we report a case of pancreaticoduodenectomy after esophagectomy with concomitant CAS.

## Case presentation

**Fig. 1:**
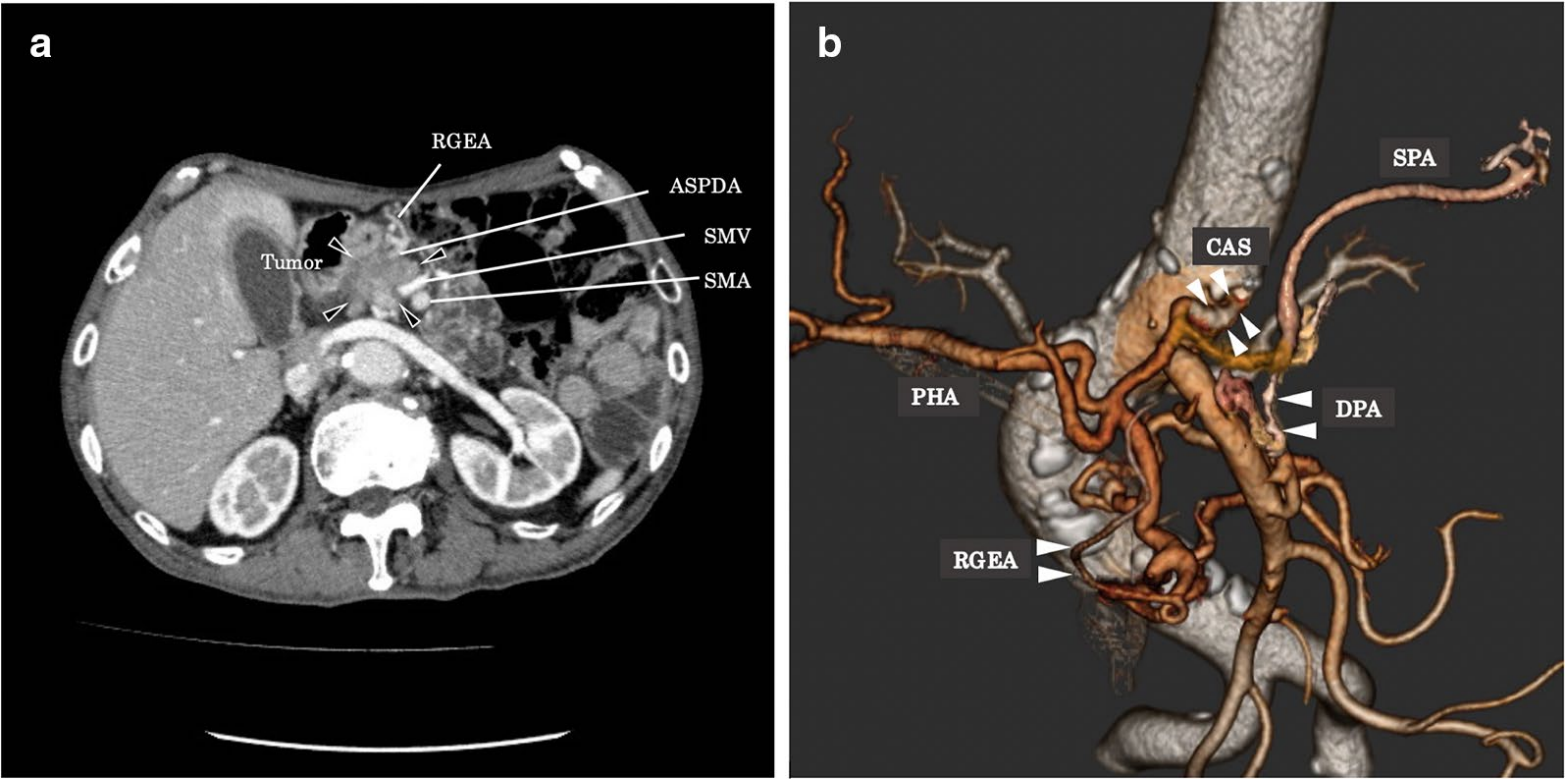
Axial view and three-dimensional reconstruction using a preoperative abdominal contrast-enhanced computed tomography. **a** Iso-dense mass (arrowheads) in the pancreatic head with attachment on the anterior superior pancreaticoduodenal artery (ASPDA). **b** Celiac artery stenosis (CAS) and branch from the superior mesenteric artery (SMA) to the splenic artery (SPA) via the dorsal pancreatic artery (DPA) before pancreaticoduodenectomy

A 76-year-old man had follow-up at our hospital after esophagectomy with retrosternal gastric conduit reconstruction for esophageal cancer, which had been performed 8 years earlier. Multi-detector computed tomography (MDCT) revealed a low-dense mass of 24 × 20 mm in the pancreatic head and infiltration to the anterior superior pancreaticoduodenal artery (ASPDA). The tumor also had attachment to the superior mesenteric vein (SMV) of less than 180 degrees (Fig. 1a). The gastric conduit was receiving its blood supply mainly from the RGEA, according to the arterial phase of dynamic CT. The RGEA was involved in the tumor. Furthermore, in this case, the celiac axis had severe stenosis. There was considerable calcification around the stenosis of the celiac artery; therefore, the cause of the stenosis was suspected to be arteriosclerosis. Although the arterial pathway between the proper hepatic artery (PHA) and the SMA were developed through the GDA, we identified well-developed collateral flow from the SMA to the splenic artery (SPA) via the DPA (Fig. 1b). Tumor markers were elevated, with a CEA of 5.6 ng/ml, and CA19-9 of 706 U/ml. Endoscopic ultrasound-guided fine-needle aspiration (EUS-FNA) revealed adenocarcinoma. The patient was diagnosed with advanced pancreatic head cancer as T2, N1, M0, and Stage IIB, according to the UICC-TNM classification, 8th Edition [11]. The tumor had no contact to SMA nor celiac axis, and contact to SMV was less than 180 degrees. According to NCCN guidelines, the resectability status would be resectable. However, we judged this case as unresectable due to the infiltration of the RGEA, because only this artery supplied arterial blood flow to the gastric conduit. Therefore, the patient received chemotherapy (nab-paclitaxel plus gemcitabine for 14 cycles). In our institution, we discuss the indication of conversion surgery after chemotherapy in case-by-case situation. In this case after 11 months of chemotherapy, we planned to perform a pancreaticoduodenectomy, because there was neither progression of the tumor nor distant metastases, CEA decreased to 5.0 ng/ml and CA19-9 to 344 U/ml, and the patient strongly requested to receive the operation. For CAS, before performing a pancreaticoduodenectomy, we tried inserting an endovascular stent for the purpose of securing blood flow of the PHA when dissecting the GDA during pancreaticoduodenectomy. We failed to place the stent due to severe stenosis, but confirmed blood flow from the SMA to the SPA via the DPA, which was detected at MDCT (Fig. 1b).

**Fig. 2:**
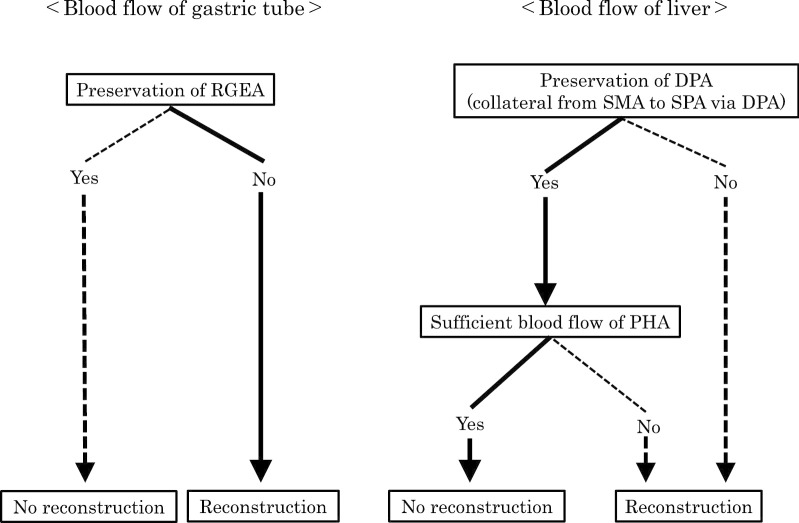
Operational strategy with celiac axis stenosis after esophagectomy. The continuous line denotes the flow of the actual operation and the dotted line denotes the flow of alternative strategies

Next, we planned to reconstruct the RGEA using the middle colic artery (MCA) during pancreaticoduodenectomy for preserving blood flow of the gastric conduit. We also planned to revascularize the PHA using the jejunal artery of the Roux-en-Y loop in case we could not preserve the DPA, which is the collateral pathway from the SMA to the SPA, or in case of deficient blood flow in the CHA after division of the PHA or the GDA in spite of preserving the DPA (Fig. 2). If the root of the GDA is involved in the tumor, the PHA should be cut and reconstructed. If the root of the GDA is tumor-free, the GDA is ligated and divided, and should be reconstructed in case of insufficient blood flow in the PHA after clamping of the GDA.

**Fig. 3:**
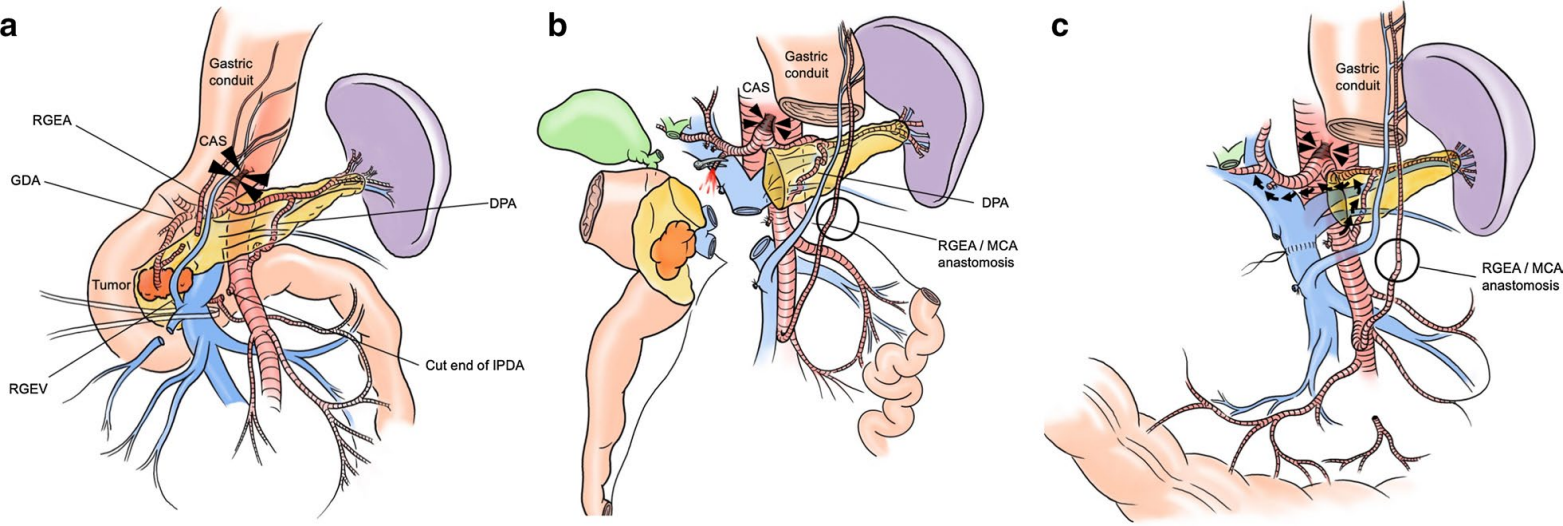
Graphic illustration of the operation record. **a** Right gastroepiploic artery (RGEA) was dissected along with the tumor, and there was no backflow from the cut end of the gastric conduit side. At the lower edge of the pancreatic body, the dorsal pancreatic artery (DPA), which communicated with the splenic artery (SPA), was identified. **b** When the gastroduodenal artery (GDA) was dissected, good blood flow from the cut end of the side of hepatic arteries was established. **c** Arterial blood flow in the proper hepatic artery (PHA) was provided from the superior mesenteric artery (SMA) to the SPA via the DPA (black arrow head)

The root of the RGEA showed tumor involvement on the preoperative CT, and the RGEA was cut at the marginal site of the remnant gastric conduit during the operation. There was no backflow from the cut end of the gastric conduit side; therefore, it was reconstructed using MCA microscopically. The RGEV could be preserved because of its distance from the tumor. Although the right gastric artery had been preserved during esophagectomy in this case, this artery was not detected at MDCT before PD. Furthermore, during PD, the right gastric artery was not palpable, and we divided this artery at the root. Using a supra-colic anterior artery-first approach [12], we dissected the trunk of the inferior pancreaticoduodenal artery (IPDA) and the first jejunal artery (J1a). The second nerve plexus of the pancreatic head (PLph-II) was dissected, preserving the nerve plexus around the SMA (PL sma). At the lower edge of the pancreatic body, the DPA, which communicated with the SPA, was identified (Fig. 3a). The gastric conduit was divided on the oral side of pyloric ring. In this case, the GDA was confirmed tumor-free intraoperatively, and blood flow after clamping of the GDA was sufficient by color Doppler ultrasonography, suggesting that the collateral circulation from the SMA to the SPA via the DPA worked sufficiently. As a result, reconstructing the PHA was unnecessary (Fig. 2). When the GDA was divided (Fig. 3b), we confirmed that the tumor had also invaded the SMV; therefore, we performed a pancreatic transection, with en bloc portal vein resection (20 mm long), and end-to-end reconstruction. The left branch of the middle colic artery (lt. MCA) was used to reconstruct the RGEA. End-to-end suturing of lt. MCA and RGEA was performed with 9–0 NYLON suture (CROWNJUN Inc., Japan) under an operating microscope (Fig. 3b, c). A retrocolic pancreaticojejunostomy, a choledochojejunostomy, and an antecolic gastrojejunostomy were performed, in this order, with a single jejunal loop. Then, a Braun anastomosis was performed between the afferent and efferent parts of the jejunal loop, which were involved in the gastrojejunostomy. Good blood flow of the remnant gastric conduit was confirmed by ICG-fluorescence imaging.

**Fig. 4:**
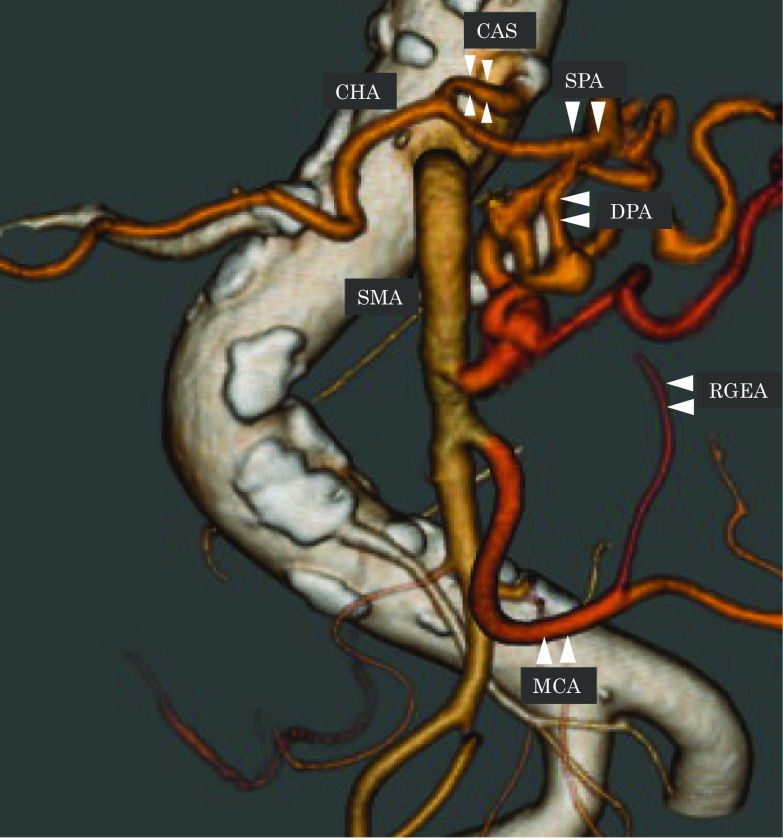
Postoperative MDCT. Postoperative enhanced CT showed that sufficient inflow was provided from the lt. MCA to the RGEA, and sufficient inflow from the SMA to the SPA via the DPA

**Fig. 5:**
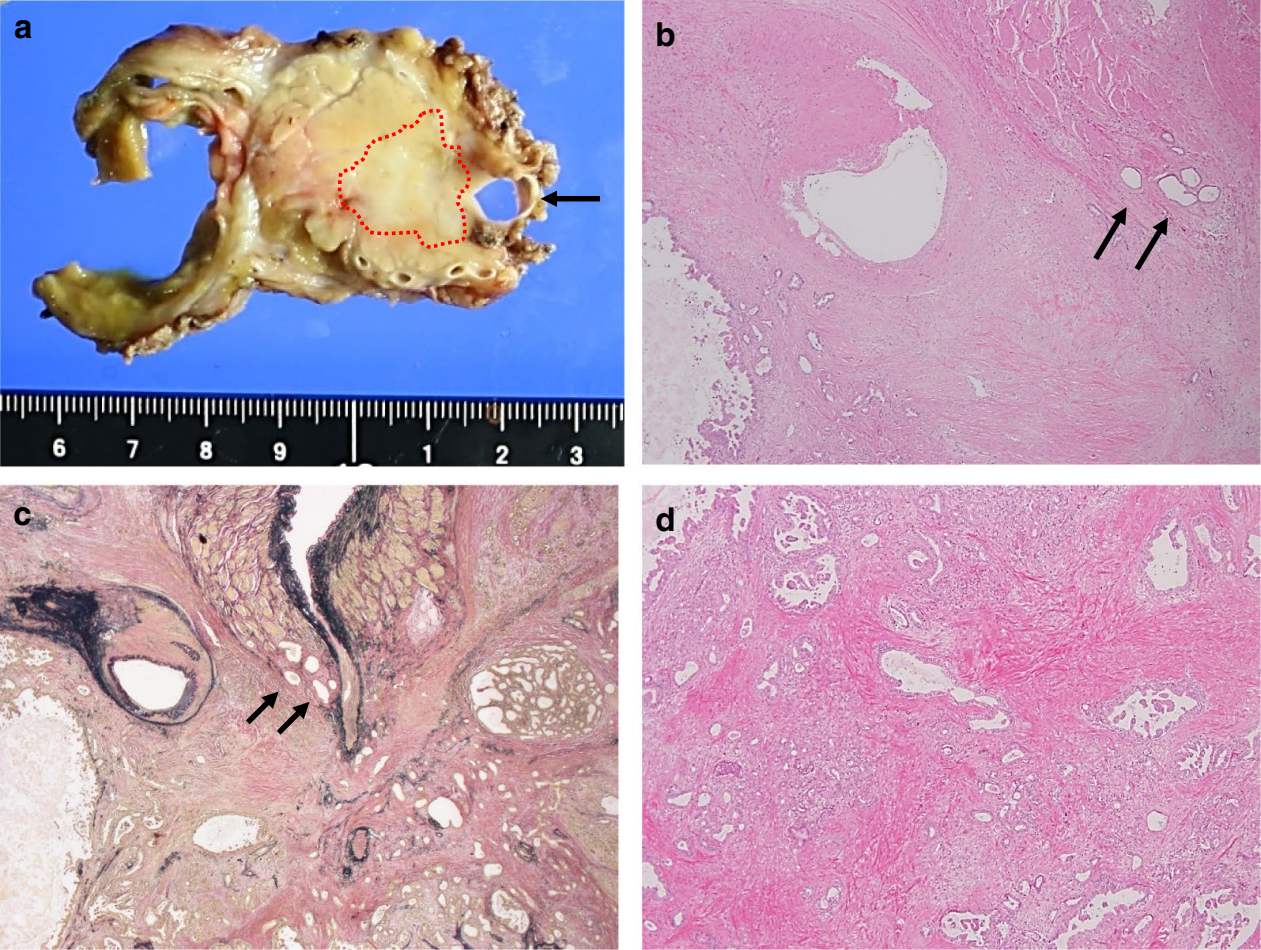
Macroscopic and microscopic view of the resected specimen. **a** The cut section of the resected specimen shows an ill-circumscribed and whitish mass (red dot) in the pancreas, invading the portal vein (arrow) and peripancreatic fat. **b**–**d** Microscopically, tubular carcinoma was seen with sclerotic stroma, invading the portal vein (arrows in **b** and **c**).[**b** and **d**: hematoxylin and eosin stain, **c**: Elastica van Gieson stain]

The total length of the operation was 598 min and the total blood loss was 350 ml. A blood transfusion with 280 ml was performed. We did not use antithrombotic therapy. A minor leakage of the gastrojejunostomy occurred in day 22. Endoscopy showed no ischemic change at the gastric conduit, and the case could be managed conservatively. We judged as functional recovery at day 43, and started S-1. However, the patient developed minor aspiration pneumonia after starting S-1, and needed swallowing rehabilitation for a while and discharged from hospital on postoperative day 90. Postoperative enhanced CT showed that sufficient inflow was provided from the lt. MCA to the RGEA, and sufficient inflow from the SMA to the SPA via the DPA (Fig. 4). Macroscopically, a 2.8 × 3.2 × 3.0 cm-sized, ill-circumscribed and whitish mass was seen, mainly located in the pancreatic head. Microscopically, the tumor was an invasive ductal carcinoma mainly composed of moderately differentiated tubular components. The carcinoma infiltrated the common bile duct, duodenum, peripancreatic fat tissue, and portal vein. Metastasis of the carcinoma to a lymph node (No. 13) located in the peripancreatic head was found. All surgical margins of the bile duct, duodenum, and pancreas were free from carcinoma. The chemotherapeutic effect determined by pathological observation was Grade IIa according to the EVANS classification, and Grade 3 according to the CAP classification [13, 14]. The overall pathological stage was T3, N1, M0, and Stage IIB, according to the UICC-TNM classification (Fig. 5a–d) [11]. Fifteen months after the surgery, the patient was alive with no recurrence.

## Discussion

Here, we report a successful resection of a locally advanced pancreatic head cancer in a patient with a history of esophagectomy and CAS. Both esophagectomy and CAS are major problems jeopardizing pancreaticoduodenectomy from the viewpoint of preserving the blood flow of the residual organs. Modern imaging technology can offer detailed information on vessel anatomy; however, a precise diagnosis of tumor infiltration is often difficult to make. We successfully performed the resection having prepared multiple surgical options to choose from based on the intraoperative findings.

In this case, the RGEA showed tumor involvement on the preoperative MDCT; therefore, there was no choice of preserving the RGEA. There have been several reports of successful pancreaticoduodenectomy associated with esophagectomy (Table 1) [15–34]. The RGEA can be preserved in patients with non-pancreatic ductal adenocarcinoma; however, in two cases, it has been described as difficult [30, 31]. Inoue et al*.* reported radical pancreaticoduodenectomy with microvascular anastomosis of the cut end of the GDA and RGEA to secure the arterial blood flow of the gastric conduit [30]. We chose MCA to reconstruct the RGEA, because we mainly use MCA to reconstruct left gastric arteries during distal pancreatectomy with celiac axis resection to preserve blood flow of the stomach, and we are experienced in the procedure [35]. Reconstruction of the RGEA using MCA has also been reported by Okochi et al.[31]. ICG-fluorescence imaging is helpful to confirm blood flow in the remnant stomach [36]. Our patient developed minor leakage at the site of the gastrojejunostomy. It was thought that the anastomotic leakage was not due to ischemia, but because the gastric conduit was drawn into the vertical space creating continuous tension in the anastomosis. The postoperative CT revealed patency of MCA-RGEA anastomosis, and upper gastroscopy revealed no ischemic change around the leaking point or specific mucosal changes associated with ischemic gastropathy [37].

**Table 1:** Pancreaticoduodenectomy after esophagectomy with a gastric conduit

Author	Year	Age	Sex	Diagnosis	Interval between esophagectomy and PD (month)	Operation	Preservation	Reconstruction	Operation time (min)	Blood loss (ml)	Complication	Recurrence /RFS (month)
Maeta [15]	1994	63	M	Pancreatic head carcinoma	72	PD	RGEA and V	None	–	–	None	None/12
Watanabe [16]	1995	65	M	Pancreatic metastasis of esophageal carcinoma	18	PD	RGEA and V	None	–	–	None	–
Hamaji [17]	1999	67	M	Gastric tube carcinoma	240	PD	None	None	–	–	Delayed oral intake	None/12
Kaneko [18]	2000	71	M	IPMC of the pancreatic head	108	PD	RGEA	None	–	–	Pancreatic fistula	None/11
Toyoda [19]	2001	75	F	Carcinoma of the ampulla of Vater	132	PPPD	RGEA	None	–	–	None	None/6
Kurosaki [20]	2003	58	M	Bile duct carcinoma	60	PPPD	RGEA and V	None	–	–	None	–
Uehara [21]	2004	57	M	IPMN of the pancreatic head	24	PD	RGEA and V	None	–	–	None	–
Tanaka [22]	2005	69	M	Carcinoma of the ampulla of Vater	14	PPPD	RGEA and V	None	555	425	None	None/36
Ikeda [23]	2006	63	M	Pancreatic head carcinoma	120	PD	RGEA and V	None	600	1080	None	None/12
Tobita [24]	2009	81	M	IPMN of the pancreatic head	72	DPPHR	RGEA and V	None	–	–	None	None/48
Hatori [25]	2009	70	M	Pancreatic head carcinoma	96	PD	RGEA and V	None	–	–	None	Yes/10
Yada [26]	2010	77	M	Duodenal carcinoma	204	PD	RGEA and V	None	–	–	None	None/18
Ando [27]	2010	67	M	Carcinoma of the ampulla of Vater	60	PPPD	RGEA and V	None	277	445	None	None/36
Addeo [28]	2011	73	M	Pancreatic metastasis of renal cell carcinoma	72	PPPD	RGEA and V	None	300	300	Pancreatic fistula	–
Fragulidis [29]	2011	50	M	Pancreatic head carcinoma	156	PPPD	RGEA	None	420	800	None	None/6
Inoue [30]	2014	72	M	Pancreatic head carcinoma	120	PD	Reconstruction	GDA and RGEA/RGEV and LRV	863	3000	None	None/6
Okochi [31]	2015	70	M	Pancreatic head carcinoma	60	PD	Reconstruction	MCA and RGEA	–	–	–	None/8
Orii [32]	2019	79	M	Pancreatic head carcinoma	60	PPPD	RGEA and V	None	–	–	None	None/63
Izumi [33]	2019	78	M	Pancreatic head carcinoma	84	PD	RGEA and V	None	492	652	None	None/5
Honig [34]	2020	72	M	Pancreatic head carcinoma	132	PPPD	RGEA and V	None	–	–	Duodenojejunostomy leak	–
Ours	2020	76	M	Pancreatic head carcinoma	96	PD	RGEV Reconstruction	MCA and RGEA	598	350	Gastrojejunostomy leak	None/15

*DPPHR* duodenum-preserving pancreatic head resection, *GDA* gastroduodenal artery, *IPMC* intraduct papillary mucinous carcinoma, *IPMN* intraduct papillary mucinous neoplasm, *LRV* left renal vein, *MCA* middle colic, *PD* pancreaticoduodenectomy, *PPPD* pylorus-preserving pancreaticoduodenectomy, *RFS* recurrence-free survival, *RGEA* right gastroepiploic artery, *V* right gastroepiploic vein

CAS was another problem in our case. There are many reports of pancreaticoduodenectomy with CAS. Causes of CAS could be median arcuate ligament syndrome (MALS) or atherosclerosis, which remain controversial [3, 38]. At first, we tried to improve blood flow by placing a vascular stent as reported by Sakorafas et al. [39]. Due to the severe stenosis, it failed to function. CAS results in the development of major collateral pathways (GDA or DPA) that arise from the SMA, resulting in the feeding of the CHA branches through retrograde flow via the GDA or the arc of Buhler [8, 9, 39, 40]. However, in patients with CAS who have developed a collateral pathway through the GDA that is sacrificed during pancreaticoduodenectomy, we must consider how to preserve the arterial flow of the liver. To prevent ischemia after pancreaticoduodenectomy, interventional radiology, arterial reconstruction, and median arcuate ligament division surgery have been considered [3, 10, 39, 41–45]. In contrast, Oikawa et al*.* reported a procedure similar to the present case which identified well-developed collateral circulation between the SMA and the SPA through the DPA and dissected GDA with any preoperative intervention or reconstruction in patients with CAS [46]. In our case, we identified well-developed blood flow from the SMA to the SPA via the DPA and successfully preserved blood flow without reconstructing the PHA. Some investigators have argued that the division of GDA during pancreaticoduodenectomy in patients with CAS does not always result in ischemic complications of the upper abdominal organs, reporting that only 13–17% of patients with CAS required arterial reconstruction during pancreaticoduodenectomy, because abundant collateral circulation beyond the pancreatic head arcade might develop between the CA tributaries and SMA tributaries [1–3]. We had planned to revascularize the PHA using the jejunal artery of the Roux-en-Y loop in the case of deficient blood flow in the PHA after dissecting the GDA (Fig. 2). Fortunately, based on the intraoperative findings, we were able to preserve blood flow in the hepatic artery without reconstruction due to the sufficient collateral blood flow from the SMA to the PHA via the DPA.

## Conclusion

In conclusion, we report a successful pancreaticoduodenectomy for pancreatic head cancer in a patient with a history of esophageal resection and CAS. Both history of esophageal resection and CAS are major problem in pancreaticoduodenectomy; therefore, careful planning based on the MDCT helps ensure a successful surgery.

## Data Availability

Not applicable.

## Abbreviations

ASPDA: Anterior superior pancreaticoduodenal artery
CAS: Celiac axis stenosis
CHA: Common hepatic artery
DPA: Dorsal pancreatic artery
EUS-FNA: Endoscopic ultrasound-guided fine-needle aspiration
GDA: Gastroduodenal artery
IPDA: Inferior pancreaticoduodenal artery
JA1: First jejunal artery
MALS: Median arcuate ligament syndrome
MCA: Middle colic artery
MDCT: Multi-detector computed tomography
PHA: Proper hepatic artery
plPh-II: Second nerve plexus of the pancreas head
pl-SMA: Nerve plexus around the SMA
RGEA: Right gastroepiploic artery
RGEV: Right gastroepiploic vein
SMA: Superior mesenteric artery
SMV: Superior mesenteric vein
SPA: Splenic pancreatic artery
